# Prevalence and genetic diversity of *Rhodococcus equi* in wild boars (*Sus scrofa)*, roe deer (*Capreolus capreolus*) and red deer (*Cervus elaphus*) in Poland

**DOI:** 10.1186/s12866-015-0445-1

**Published:** 2015-05-22

**Authors:** Lucjan Witkowski, Magdalena Rzewuska, Agata Anna Cisek, Dorota Chrobak-Chmiel, Magdalena Kizerwetter-Świda, Michał Czopowicz, Mirosław Welz, Jerzy Kita

**Affiliations:** Laboratory of Veterinary Epidemiology and Economics, Faculty of Veterinary Medicine, Warsaw University of Life Sciences, Nowoursynowska 159c, 02-776 Warsaw, Poland; Department of Preclinical Sciences, Faculty of Veterinary Medicine, Warsaw University of Life Sciences, Ciszewskiego 8, 02-786 Warsaw, Poland; Voivodeship Veterinary Inspectorate in Krosno, ks. Piotra Ściegiennego 6 A, 38-400 Krosno, Poland

**Keywords:** Rhodococcosis, Epidemiology, PFGE, Wildlife, Wild ruminant

## Abstract

**Background:**

*Rhodococcus equi* is now considered an emerging zoonotic pathogen. Sources and routes of human infection remain unclear but foodborne transmission seems to be the most probable way. Strains of pig or bovine type are most often isolated from human cases and moreover *R. equi* is present in submaxillary lymph nodes of apparently healthy pigs and wild boars intended for human consumption. The aim of this study was to estimate the prevalence of *R. equi* in submaxillary lymph nodes in wild boars, roe deer and red deer.

**Results:**

Samples were collected from 936 animals and 27 *R. equi* strains were isolated, from 5.1 % of wild boars (23/452), 0.7 % of red deer (2/272) and 0.9 % of roe deer (2/212). Genetic diversity of all 27 isolates was studied using *Vsp*I-PFGE method, resulting in the detection of 25 PFGE patterns and four PFGE clusters. PFGE patterns of the isolates were compared with virulence plasmid types and no concordance was observed.

**Conclusions:**

*R. equi* was present in wild animal tissues and consumption of the game may be a potential source of *R. equi* infection for humans. To the authors’ best knowledge, this is the first epidemiological report of *R. equi* prevalence in tissues of roe deer and red deer. However, risk associated with wild ruminant consumption seems marginal.

Investigation of *R. equi* transmission between animals and humans based exclusively on types of virulence plasmids seems to be insufficient to identify sources of *R. equi* infection for people.

## Background

*Rhodococcus equi* is a Gram-positive bacterium present in the intestinal microflora of grazing farm animals and wild animals such as deer, wild boars and others, as well as widespread in their environment [1-5]*. Rhodococcus equi* can cause diseases in various animals. However, only foals up to six months of age have a unique susceptibility to clinical disease and it is a major concern to the equine breeding industry [6]. Clinical form of this disease known as rhodococcosis mostly manifesting itself with abscesses or lymphadenitis has been also reported occasionally in other farm animals. In pigs *R. equi* causes lymphadenitis [7, 8] but it is also present in lymph nodes of healthy animals intended for human consumption [3, 9-13]. In ruminants the disease has been described quite rarely and most often in goats [14]. In cattle *R. equi* was isolated from purulent lesions in various tissues [10, 15-17]. Clinical cases of pyogranulomatous skin disease and pneumonia associated with *R. equi* have been rarely observed in cats and dogs [18].

The first report of *R. equi* isolation from tissues of wildlife was published in 2008 [19]. The presence of *R. equi* was demonstrated in 12.4 % of the submaxillary lymph node samples collected from wild boars’ carcasses. Afterwards, *R. equi* was isolated from 6.6 % mesenteric and submaxillary lymph nodes with lymphadenitis in wild boars in Brazil, but the bacteria were not detected in lymph nodes without lymphadenitis [12, 13]. Then, very high *R. equi* prevalence of 52 % was noticed in submaxillary lymph nodes of wild boars in Japan [20]. Recently, *R. equi* isolation from lymphatic tissues has also been described in Poland [21]. Furthermore, the case of bronchopneumonia in wild boars caused by *R. equi* was reported in 2013 in Brazil [22].

Little is known about the occurrence of *R. equi* in wild ruminants. *R. equi* were isolated from feces of deer in New Zealand [1] and African indigenous ruminants [2]. The first isolation of *R. equi* from tissues of healthy roe deer and red deer was reported in 2014 [21]. Infection in wild ruminants associated with *R. equi*, has been thus far described only in American bison (*Bison bison*) with paratuberculosis where this bacterium was isolated from caseous necrotic lesions together with *Mycobacterium avium* subsp. *paratuberculosis* [23].

An increasing number of *R. equi* infections in humans have been reported in last decades and *R. equi* is now considered an emerging zoonotic pathogen [24, 25]. Even though sources and routes of human infection remain unclear, a foodborne transmission seems to be the most probable way especially by contact and consumption of raw and undercooked meat [24-26].

The virulence of *R. equi* is determined by the virulence associated proteins (Vaps). The equine *R. equi* isolates carry the *vapA* gene encoding virulence-associated 15–17-kDa protein (VapA), swine and cattle isolates harbor mainly the *vapB* gene of the virulence-associated 20-kDa protein (VapB), and cattle isolates can carry the *vapN* gene [27]. These genes are placed on virulence-associated plasmids (VAPs), VAPA, VAPB, and VAPN, respectively. The “TRAVAP” typing scheme classifies *R. equi* isolates into 4 categories: *traA*^*+*^*/A*^*+*^*B*^*−*^ “horse-type”, *traA*^*+*^*/A*^*−*^*B*^*+*^ “pig-type”, *traA*^*+*^*/AB*^*−*^ “bovine-type” and *traA*^*−*^*/AB*^*−*^ plasmid-less type. Avirulent strains showing no evidence of plasmids are widespread mainly in soil. From clinical cases of infections in humans, the strains of pig or bovine type have been isolated more often than avirulent or equine strains [4, 26, 27]. Analysis of restriction enzyme digestion patterns revealed several distinct VAPA types and over 20 types of VAPB [4, 12]. Furthermore, there is a geographical diversity among human and animal isolates e.g. VAPB type 5 is predominant in Europe [21], type 8 in South America [12], type 1 and 2 in Asia [3, 20].

The aim of this study was to estimate the prevalence of *R. equi* in wild boars, roe deer and red deer carcasses intended for human consumption in Poland and to determine the genetic diversity of the isolates.

## Results

A total number of 936 lymph nodes samples were evaluated; 452 from wild boars, 272 from red deer and 212 from roe deer. The animals were hunted in various regions, in 12 of 16 voivodships of Poland. In collected lymph nodes from wild boars the purulent lesions were observed in 8.4 % (CI 95: 6.2-11.3 %) of samples. Size of abscesses varied from 1 to 50 mm in diameter. No lesions were observed in any samples from wild ruminants. *R. equi* was isolated from lymph nodes collected from 23 wild boars (5.1; CI 95: 3.4, 7.5 %), 2 red deer (0.7; CI 95: 0.2, 2.6 %) and 2 roe deer (0.9; CI 95: 0.2, 3.6 %). All 27 *R. equi* isolates were recovered from tissues without any anatomopathological lesions.

**Fig. 1:**
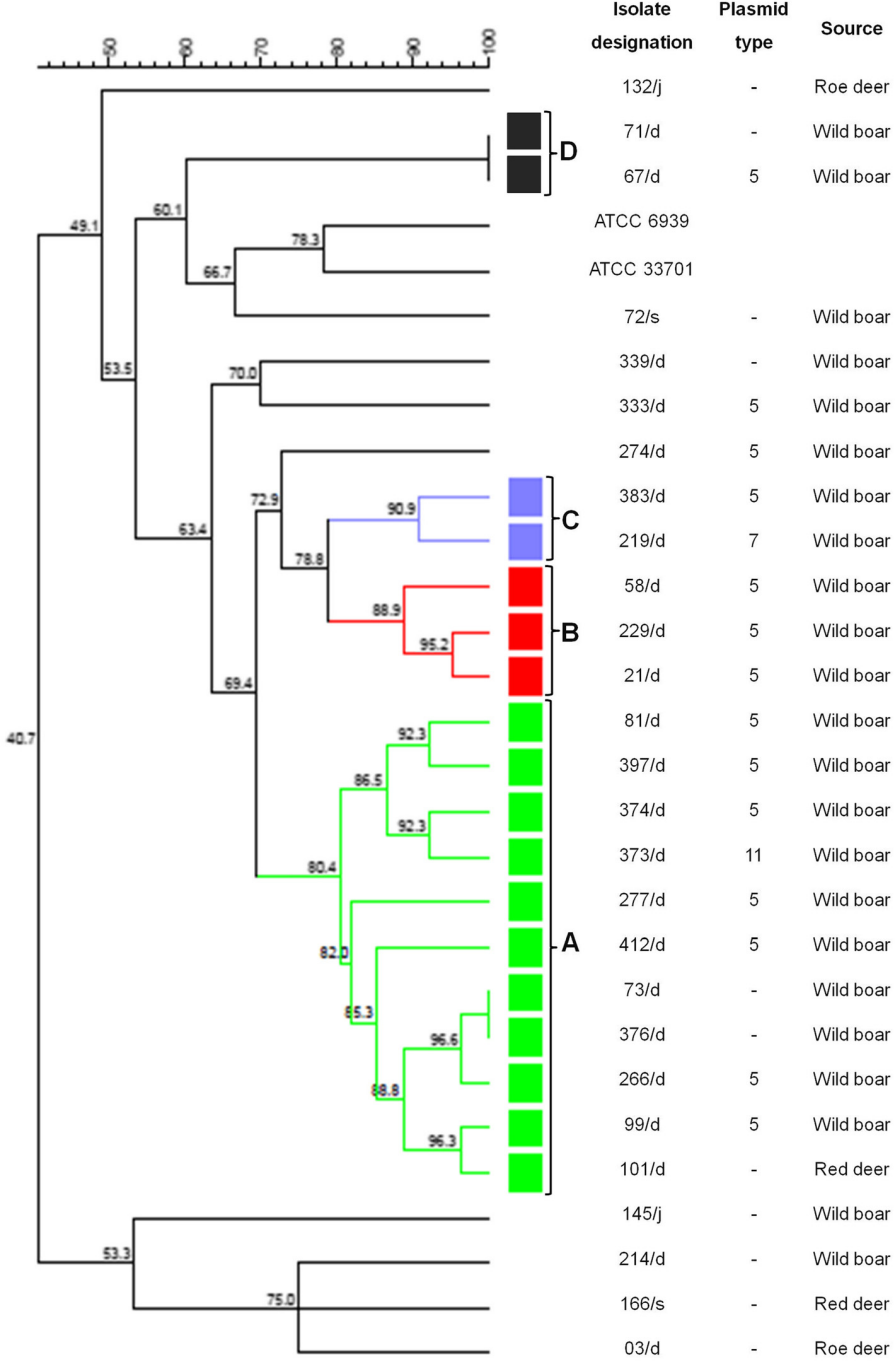
Genotyping of 27 *Rhodococcus equi* strains with *Vsp*I-PFGE method

Phenotypic characterization, virulence genotypes and plasmid profile of these isolates was published previously [21] and allowed to compare these features with PFGE results. All 27 isolates were subjected to genotyping with PFGE. The isolates were typeable with *Vsp*I-PFGE, resulting in the detection of 25 PFGE patterns and four PFGE clusters (A, B, C, D) (Fig. 1). The isolates were considered to be closely related above 80 % of homology and assigned to the same PFGE cluster. The major cluster (A) was shared by 11 isolates from wild boars, cluster B was shared by three isolates from wild boars and clusters C and D consisted of two isolates each from wild boars. The remaining 12 isolates exhibited different pulsed-field patterns. Unfortunately, the data about animals were not available in all cases and it did not allow identification of each sample with place of origin. In PFGE cluster A nine isolates harbored plasmid type 5, one type 7 and one type 11. In cluster B two isolates contained plasmid type 5 and one isolate was plasmid-less. Interestingly, two strains in cluster A and two in D isolated from wild boars were indistinguishable and had the same PFGE pattern. Both indistinguishable isolates in cluster A carried type 5 plasmid and were recovered from animals captured in the same forest. Interestingly, one strain in cluster D carried plasmid type 5 but the second was avirulent. Unfortunately, in this case origin of the strain was unknown.

## Discussion

Our results confirmed occurrence of *R. equi* in submaxillary lymph nodes of apparently healthy wild boars. The prevalence of 5.1 % in the studied wild boar population in Poland appears low compared to the prevalence of 12.4 % in Hungary [19], and much lower than the prevalence of 52 % in Japan [20]. Interestingly, the results are similar to data from Brazil (6.6 %) [13]. However, comparison of the results needs to be done with caution. In the Brazilian study *R. equi* was isolated only from wild boar lymph nodes with lymphadenitis and all investigated lymph nodes without lesions were negative. Moreover, contrary to Poland, wild boars in Brazil are not wildlife but animals exotic for local fauna, kept on commercial farms in semi-extensive conditions. Clinical cases of pulmonary infection caused by *R. equi* were described in wild boars only in Brazil [22].

In this investigation purulent lesions in lymph nodes were found in 8.4 % of samples, but they were not associated with *R. equi* infection. In comparison, in slaughtered farm pigs granulomatous lesions in the submaxillary lymph nodes caused by various pathogens were observed in 0.75 % of animals [7]. Given that pigs are usually kept in buildings, feed with commercial feed, and treated with antibiotics, risk of bacterial infection is lower compared to wild animals in the natural environment.

Regarding that the methodology of *R. equi* isolation was similar in all the aforementioned studies it is interesting why *R. equi* prevalence among wild boars from different countries varied so much. Relatively wide differences in the prevalence of *R. equi* between studies were also observed in pigs intended for human consumption where it varied from 0.0 to 21.7 % [3, 9-13]. It was found that *R. equi* prevalence in young wild boars could be higher than in older ones [20], also the prevalence in younger cattle was higher than in older cows [15]. It may suggest that older animals are more resistant to *R. equi* infection or there are other not known predisposing factors like e.g. immunocompetence of individual animal. Study from Japan showed that the prevalence of *R. equi* in wild boars was the same in various regions [20] but factors like wide dissemination of *R. equi* in the environment, density of animal population, their diet, individual susceptibility to the infection and other may have played a role.

To date, the prevalence of *R. equi* in tissues of roe deer and red deer has not been investigated. This bacterium was detected previously only in feces of deer in New Zealand [1] and other wild ruminants in Africa [2]. The *R. equi* isolation from tissues was also reported in 5.7 % of American bison with paratuberculosis [23]. Recently, *R. equi* isolation from submaxillary lymph nodes of healthy roe deer and red deer has been described in Poland [21] and in this study the epidemiological data on this material are presented. Very low prevalence of avirulent, environmental strains of *R. equi* (less than one per cent in red deer and roe deer), and lack of tissue lesions suggest that it should be interpreted as an accidental carriage of the pathogen. Generally, ruminants seem to be relatively resistant to *R. equi* infection. In population of slaughtered cattle the prevalence of *R. equi* was very low (0.008 %) [15]. However, clinical disease has been reported sporadically in domestic goats [14] and cattle, but all descriptions of *R. equi* isolation from cattle concern animals with purulent lesions or suspected of *Mycobacterium* spp. infection [10, 15-17]. *R. equi* was detected alone or together with *Mycobacterium* spp. in granulomas in 3.9 % of bovine lymph node samples in Ireland [15], 4 % in Algeria [16] and 19.5 % in Czech Republic [10]. These isolates were obtained mainly from retropharyngeal, bronchial and mediastinal lymph nodes which are most common sites of tuberculosis lesions. It was also suggested that *Mycobacterium* spp. infection could predispose to *R. equi* infection [23]. Purulent lesions in wildlife associated with infections with other Gram-positive bacteria including *Mycobacterium* spp*.* or *Trueperella* (*Arcanobacterium) pyogenes* [23, 28, 29] are more frequently described. However, *R. equi* as a common soil organism of unknown clinical importance is rarely taken into consideration as a potential pathogen. Presented results suggest that infection or co-infection with *R. equi* should be more often considered as a differential diagnosis of purulent lesions in wildlife.

Genotypic method often used in the epidemiological investigations of genetic relationships between *R. equi* isolates was PFGE, however, results were inconclusive [5, 11, 17, 30-34]. In the case of *R. equi* infection in horses it was shown that many various strains were widespread in horse population but individual farms tended to harbor a particular single strain [30-32]. However, another study revealed high genetic variability not only among isolates from various countries but also on farms [33]. Furthermore, using other technique (repetitive sequence-based PCR) it was shown that one foal could be infected with multiple *R. equi* strains [34]. Application of PFGE in the investigation of swine *R. equi* strains showed high genetic diversity of strains not only between farms but also between individual animals on one farm [11]. It was suggested that infection was not presumably transmitted among animals in the herd but the environment could have been a source of infection. Interestingly, in other study cattle strains isolated from various farms had the same restriction pattern [17]. Unfortunately, in our study complete information about the origin of each positive animal was not available. Comparison of PFGE patterns and plasmid type in this study confirmed previous observation in pigs [11], horses [32] and cattle [5] where *R. equi* isolates containing the same plasmid type revealed different PFGE patterns and vice versa isolates with identical PFGE patterns contained different virulence plasmids or were plasmid-less.

*R. equi* is a zoonotic pathogen. Even though sources and routes of infection in humans remain unclear, exposure to animals or their environment may play a role in some cases of infection in humans [24-26]. *R. equi* is thought to be acquired by inhalation from soil, inoculation into wound or mucous membranes, or ingestion and passage through the alimentary tract. These results confirmed the presence of *R. equi* in tissues of wild animals intended for human consumption. It could be a source of human infection. This study showed that *R. equi* strains sharing the same plasmid type had different PFGE patterns. Thus, investigation of *R. equi* transmission between animals and humans based exclusively on types of virulence plasmids seems to be insufficient to identify sources of *R. equi* infection for people. For this purpose total genomic DNA comparison of *R. equi* strains obtained from clinical cases of infection in humans with isolates from wildlife, farm animals and environment is warranted.

## Conclusions

*R. equi* was present in wild animal tissues and consumption of the game may be a potential source of *R. equi* infection for humans. To the authors’ best knowledge, this is the first epidemiological report of *R. equi* prevalence in tissues of roe deer and red deer. However, risk associated with wild ruminant consumption seems marginal.

Investigation of *R. equi* transmission between animals and humans based exclusively on types of virulence plasmids seems to be insufficient to identify sources of *R. equi* infection for people.

## Materials and Methods

The study was approved by the 3rd Local Commission for Ethics in Animal Experiments (Decision No. 44/2009). Population of studied wild animals was estimated based on the data of the Polish Hunting Association monitoring in the 2009/2010 season [35]. The population of wild boars, roe deer and red deer counted 250 000, 757 000 and 145 000 individuals, respectively. During this season 197 000 wild boars, 162 000 roe deer and 41 100 red deer were hunted. Required sample size (n) was determined for each animal species according to the following formula: n = [1.96^2^ × P_exp_ × (1 – P_exp_)] / d^2^ assuming desired absolute precision (d) of 5 % in wild boars, and 10 % in roe and red deer, expected prevalence (P_exp_) of 50 % and 95 % level of confidence [36]. The calculations were performed using Win Episcope 2.0. Minimum sample size was 385 and 97 animals for wild boars and deer, respectively. 95 % confidence intervals were calculated using Wilson score method.

According to the Polish regulation all carcasses of hunted animals are collected by a few companies. Submaxillary lymph nodes were obtained from wild boars, red deer and roe deer carcasses collected in facilities belonging to 2 companies during seasons 2009/2010 and 2010/2011. All studied samples were obtained from carcasses accepted for human consumption and stored in -20 °C for further investigation. Refrozen lymph nodes were cut into small pieces using sterile scissors. Then, one gram of tissue was added to 3 ml of sterile 0.9 % saline and was homogenized using PRO200 homogenizer Multi-Gen 7 (PRO Scientific Inc., USA). Finally, 100 μl of homogenized tissue was cultured to selective medium. *R. equi* isolation, phenotypic and genotypic identification of isolates was conducted as described previously [21]. Briefly, for the bacteria isolation modified CAZ-NB medium was used, biochemical properties were determined in API Coryne test (bioMerieux, France) and the presence of “equi factor” was studied in CAMP test. Isolate identification was confirmed by MALDI-TOF MS using VITEK MS (bioMerieux, France). The presence of four *R. equi* genes, *choE, traA, vapA* and *vapB* was determined by PCR.

PFGE was performed as previously described [31] with minor modifications. Briefly, the overnight *R. equi* cultures in BHI medium (bioMérieux, France) enriched with 0.4 % glucose, 1 % glycerol and 0.2 % Tween 85 were adjusted to OD_600_ 0.65 and the cells were incorporated into 1.5 % (w/v) agarose discs (SeaKem Gold, Lonza, Switzerland). After 18 h of lysis with lysozyme (20 mg/ml, SIGMA, Germany) and RNase (50 μg/ml, Fermentas, Lithuania) at 37 °C, discs were incubated with proteinase K (300 μg/ml, A&A Biotechnology, Poland) overnight at 50 °C. Then DNA in agarose discs was digested with *Vsp*I (10 U/μl; Fermentas, Lithuania) overnight at 37 °C. The restriction fragments were separated by clamped homogenous electric field electrophoresis with a CHEF-DR II System (Bio-Rad Laboratories, USA) in a 1.1 % (w/v) agarose gel using the following conditions: running time 22 h, temperature 14 °C; voltage gradient 200 V; included angle 120°. During the first run (7 h), an initial pulse time of 6 s and a final pulse time of 15 s were used, and during the second run (15 h), an initial pulse time of 23 s and a final pulse time of 40 s were used. Lambda ladder PFGE Marker (BioLabs, New England) was selected for molecular size estimation. The gel was stained with ethidium bromide (0.5 μg/ml) for 30 min at room temperature with gentle shaking, then destained in distilled water for 30 min, documented and analysed by a VersaDoc Imaging System (model 1000) and a Quantity One software (version 4.4.0) (BioRad, USA). Afterwards, the gel images were analyzed by Gel Compar II version 4.6 (Applied Maths, Belgium) and a cluster analysis was performed by UPGMA using dice similarity coefficient with optimization set at 1 % and position tolerance at 1.5 %. The isolates were clustered using an 80 % homology cut-off, above which the isolates were considered to be closely related and assigned to the same PFGE cluster [37]. The reference *R. equi* strains ATCC 6939 and ATCC 33701 were used in the study as a control.
